# Bi-Functionalization of a Calcium Phosphate-Coated Titanium Surface with Slow-Release Simvastatin and Metronidazole to Provide Antibacterial Activities and Pro-Osteodifferentiation Capabilities

**DOI:** 10.1371/journal.pone.0097741

**Published:** 2014-05-20

**Authors:** Yunsong Liu, Xiao Zhang, Yang Liu, Xiaoxiao Jin, Cong Fan, Hongqiang Ye, Meng’en Ou, Longwei Lv, Gang Wu, Yongsheng Zhou

**Affiliations:** 1 Department of Prosthodontics, School and Hospital of Stomatology, Peking University, Beijing, China; 2 National Engineering Laboratory for Digital and Material Technology of Stomatology, Beijing, China; 3 Department of Orthodontics, School and Hospital of Stomatology, Peking University, Beijing, China; 4 Department of Oral Implantology and Prosthetic Dentistry, Academic Centre for Dentristry Amsterdam (ACTA), Research Institute MOVE, VU University and University of Amsterdam, Amsterdam, The Netherlands; 5 Central Laboratory, School and Hospital of Stomatology, Peking University, Beijing, China; Instituto de Engenharia Biomédica, University of Porto, Portugal

## Abstract

Coating the surface of titanium implants or other bone graft substitute materials with calcium phosphate (Ca-P) crystals is an effective way to enhance the osteoconduction of the implants. Ca-P coating alone cannot confer pro-osteodifferentiation and antibacterial capabilities on implants; however, it can serve as a carrier for biological agents which could improve the performance of implants and bone substitutes. Here, we constructed a novel, bi-functional Ca-P coating with combined pro-osteodifferentiation and antibacterial capabilities. Different concentrations of metronidazole (MNZ) and simvastatin (SIM) were integrated into biomimetic Ca-P coatings on the surface of titanium disks. The biological effects of this bi-functional biomimetic coating on human bone marrow mesenchymal stem cells (hBMMSCs), human adipose derived stromal cells (hASCs), and *Porphyromonas gingivalis* were assessed *in vitro*. We observed that Ca-P coatings loaded with both SIM and MNZ display favorable release kinetics without affecting cell proliferation or attachment. In the inhibition zone test, we found that the bi-functional coating showed lasting antibacterial effects when incubated with *Porphyromonas gingivalis* for 2 and 4 days. Moreover, the osteodifferentiation of hBMMSCs and hASCs were increased when cultured on this bi-functional coating for 7 and 14 days. Both drugs were loaded onto the Ca-P coating at specific concentrations (10^−5^ M SIM; 10^−2^ M MNZ) to achieve optimal release kinetics. Considering the safety, stability and low cost of SIM and MNZ, this novel bi-functional Ca-P coating technique represents a promising method to improve the performance of metal implants or other bone substitute materials, and can theoretically be easily translated to clinical applications.

## Introduction

Titanium (Ti) implants are widely used for the fixation of long bone non-unions, the stabilization of spinal fractures, and the restoration of missing teeth. However, the slow rates of metal implant-bone osseointegration as well as implant-associated infections have become the primary risk factors for patients [1]–[3].

Recently, a biphasic biomimetic calcium phosphate (Ca-P) coating technique was reported for the surface modification of Ti implants or other bone graft substitute materials [4]–[6]. Although the biomimetic Ca-P coating improves the osteoconductivity of metal implants, it does not confer osteoinductivity, which promotes the differentiation of immature progenitor cells along an osteoblastic lineage, to the implants. Moreover, further studies are necessary to improve the antibacterial capability of this Ca-P coating.

Interestingly, some recent studies [7]–[10] have reported that Ca-P coating of the implant surface can also act as a carrier for the controlled release of biological agents such as osteoinductive, antibacterial and anti-inflammatory agents. Thus, Ca-P coating could confer multi-functional capabilities to coated implants or bone graft substitute materials. However, the multifunctional potential of Ca-P coating in combination with osteoinductive and antibacterial agents has not been thoroughly investigated, nor do practical protocols currently exist that can be applied clinically to guide the preparation of multifunctional Ca-P coating on Ti implants.

Previous studies have demonstrated that simvastatin (SIM) can increase the osteogenic capability of mesenchymal stem cells (MSCs) [11], [12]. SIM has several advantages over bone morphogenetic proteins (BMPs) for use with Ca-P coatings, such as chemical stability, ease of processing, and low cost. Metronidazole (MNZ) is a commonly-used drug with stable physicochemistry and a relatively broad anti-bacterial spectrum targeted to microaerophilic and anaerobic bacteria [13], [14]. In this study, we constructed a novel, bi-functional Ca-P coating incorporating SIM and MNZ. Systematic observations of the surface characteristics of the bi-functional coatings and time-release kinetics of the incorporated agents were performed to optimize Ca-P coating. Moreover, the biological effects of this bi-functional, biomimetic coating on human mesenchymal stem cells (MSCs) and *Porphyromonas gingivalis* (oral bacterium) were assessed *in vitro*.

## Materials and Methods

### Ethics Statement

Human bone marrow mesenchymal stem cells (hBMMSCs) and human adipose derived stromal cells (hASCs) were purchased from ScienCell Company (San Diego, CA, USA). This study was approved by the Ethics Committee of the Peking University Health Science Center, Beijing, China (PKUSSIRB-2013023).

### Preparation of drug loaded Ca-P coating

All materials were purchased from Sigma-Aldrich (St. Louis, MO, USA) unless otherwise stated.

Flat, commercial, pure Ti (grade III, Beijing General Research Institute For Non-ferrous Metals, China) disks (8 mm diameter×0.8 mm thickness) were polished, sandblasted and etched (sandblast large grit and acid-etching, SLA) according to previously reported procedures [15].

Biomimetic Ca-P coating of Ti disks was performed by a biphasic Ca-P coating technique [4]. Step 1: SLA disks were immersed in a five-fold concentrated simulated body fluid (5×SBF) for 24 h at 37°C with stirring at 60 rpm. A fine, dense layer of amorphous Ca-P forms and serves as a seeding substratum for the deposition of a more substantial crystalline layer. Step 2: the crystalline layer was produced by immersing the amorphous Ca-P coated disks in a supersaturated Ca-P solution for 48 h at 37°C with shaking at 60 rpm. Finally, all the samples were washed and freeze-dried for 12 hours (MICRO MODULYO-230, Thermo Electron Corporation, USA).

Ca-P coatings were loaded with SIM as mentioned above, except that different concentrations of SIM stock solution were added to the supersaturated Ca-P solution to form a concentration gradient (10^−3^, 10^−4^ and 10^−5^ M SIM solutions) in the second step. In the same way, different doses of MNZ were added to the supersaturated Ca-P solution to form a concentration gradient (10^−2^, 10^−3^ and 10^−4^ M MNZ solutions). For the preparation of bi-functional coatings (biomimetic Ca-P coating loaded with SIM and MNZ), specific doses of SIM and MNZ were added to the same supersaturated Ca-P solution.

### Characteristics of the coating surfaces

Field emission scanning electron microscopy (FESEM, S-4800; HITACHI, Japan) and energy dispersive X-ray spectroscopy (EDS) were used to analyze the morphology and elementary components of the surface of the coatings, respectively.

### *In vitro* evaluation of the release kinetics of drug-loaded Ca-P coatings

Ca-P-coated disks loaded with SIM (10^−3^, 10^−4^ and 10^−5^ M, n = 5 for each concentration) and MNZ (10^−2^, 10^−3^ and 10^−4^ M, n = 5 for each concentration) were immersed in 200 µL PBS using a 48-well plate. Ca-P-coated disks in the absence of SIM and MNZ were used as controls. All plates were kept at 37°C with shaking at a rate of 60 rpm. At predetermined time intervals of 1, 2, 3, 4, 5, 6, 7, 10, 14, and 21 days, the SIM and MNZ contents were measured using a Multimode Plate Reader (EnSpire, PerkinElmer Co., Shelton, CT, USA) at a wavelength of 238 nm [16] and 313 nm [17].

### Inhibition zone test

In order to study the biological effects of the bi-functional coating on bacteria and MSCs, five groups of Ti disks were labeled as follows: 1. SLA Ti disk (SLA); 2. SLA disk with Ca-P coating (Ca-P); 3. SLA disk with MNZ-loaded Ca-P coating (Ca-P+MNZ); 4. SLA disk with SIM-loaded Ca-P coating (Ca-P+SIM); 5. SLA disk with SIM and MNZ-loaded Ca-P coating (Ca-P+MNZ+SIM). SLA served as a negative control group while Ca-P served as a negative coating control group.

*P. gingivalis* W83 (ATCC, Manassas, VA, USA) was used to assess the antibiotic capability of the coating in the inhibition zone test. Cultures of *P. gingivalis* were suspended in sterile liquid culture medium (distilled water containing 37 g/L Brain Heart Infusion, L-cysteine and 5 g/L Yeast Extract) and evenly distributed on the blood agar plates [18]. To test the sustainability of the antibacterial capability of the coatings in a liquid environment, all five groups of Ti disks were immersed in PBS (changed daily) for 2 and 4 days, and then tested using the inhibition zone test.

### Cell culture

Human bone marrow mesenchymal stem cells (7500, ScienCell) and human adipose derived stromal cells (7510, ScienCell) were used to assess the pro-osteodifferentiation capability of the bi-functional coating.

All cells were cultured in proliferation medium (PM) containing Dulbecco's modified Eagle's medium containing 10% fetal bovine serum (FBS), 100 U/mL penicillin G and 100 µg/mL streptomycin at 37°C in an incubator with an atmosphere comprising 95% air, 5% CO_2_ and 100% relative humidity. All cell-based experiments were repeated at least two times.

### Cell proliferation assay

Cell numbers were determined using the cell-counting kit-8 (CCK8) according to the manufacturer's instructions (Dojindo Laboratories, Kumamoto, Japan). Growth curves were drawn according to the absorbance values (mean ± SD, n = 4).

### Cell differentiation assay

Cells were seeded onto five groups of Ti disks in osteogenic medium (OM) containing 200 µM ascorbic acid, 10 mM β-glycerophosphate and 100 nM dexamethasone to induce osteogenesis. Alkaline phosphatase (ALP) activity assays were performed using an ALP kit according to the manufacturer's protocol.

### Determination the protein secretion of bone morphogenetic protein-2 (BMP-2)

Cells were seeded in 24-well plates (1×10^4^ cells/well). After 7 and 14 days of incubation, culture supernatants were collected and stored at −20°C. The amounts of BMP-2 were determined by enzyme-linked immunosorbent assay (ELISA) kits (R&D System, Minneapolis, USA) according to the manufacturer's instructions. The assay was performed in three independent experiments.

### Real-time qRT-PCR

Total RNA was extracted according to the manufacturer's protocol (Invitrogen, Carlsbad, CA, USA). One microgram aliquots of RNA were reverse-transcribed according to the manufacturer's protocol (Invitrogen). Real-time quantitative PCR assays were performed according to the manufacturer's instructions (Bio-Rad, Hercules CA, USA). The primers for Runt-related transcription factor 2 (*RUNX2*), osterix (*OSX*), and osteocalcin (*OCN*) were synthesized by Invitrogen and are listed in Table 1. Glyceraldehyde 3-phosphate dehydrogenase (*GAPDH*) was used as an internal standard.

**Table 1:** Sequences of the primers used for real-time PCR.

Genes	Forward primer	Reverse primer
*RUNX2*	TCTTAGAACAAATTCTGCCCTTT	TGCTTTGGTCTTGAAATCACA
*OSX*	CCTCCTCAGCTCACCTTCTC	GTTGGGAGCCCAAATAGAAA
*OCN*	CACTCCTCGCCCTATTGGC	CCCTCCTGCTTGGACACAAAG
*GAPDH*	ATGGGGAAGGTGAAGGTCG	GGGGTCATTGATGGCAACAATA

### Immunofluorescent staining for OCN

After 14 days of culture, the cells cultured on different groups of Ti disks were rinsed three times with PBS and the immunofluorescent staining for OCN was performed according to the manufacturer's protocol (Cell Signaling, Danvers, MA, USA). After staining for OCN, the cells were counterstained with DAPI for nuclear staining and visualized using a confocal laser scanning microscopy (CLSM, TLS SP2; Leica, Wetzlar, Germany).

### Statistical analysis

Data are expressed as the mean ± standard deviation and analyzed using SPSS software. One-way analysis of variance followed by Fisher's least significant difference test was performed. For all tests, statistical significance was accepted at *P*-values lower than 0.05.

## Results

### Morphological analysis of the coatings

**Figure 1:**
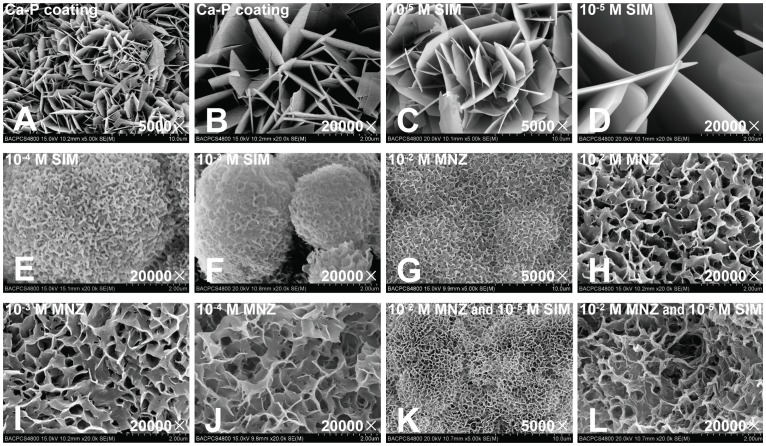
Scanning electron microscopy (SEM) observations of the Ca-P coating and drug-loaded Ca-P coating. (A, B) Ca-P coating. (C, D) Ca-P coating loaded with 10^−5^ M SIM. (E, F) Ca-P coating loaded with 10^−4^ M and 10^−3^ M SIM. (G, H) Ca-P coating loaded with 10^−2^ M MNZ. (I, J) Ca-P coating loaded with 10^−3^ M MNZ and 10^−4^ M MNZ. (K, L) Ca-P coating loaded with 10^−2^ M MNZ and 10^−5^ SIM together.

After surface treatment of the Ti disks, the biomimetic Ca-P coating was successfully deposited onto the disks using a biphasic coating technique. SEM observations showed that the Ca-P coating was entirely composed of straight, plate-like and sharp-edged crystal units, and the length of the crystal units varied between 2 and 5 µm (Fig. 1A, B).

When loaded with 10^−5^ M SIM, the morphology of the coating was similar to Ca-P alone; however, the length of the crystal units was slightly longer and varied between 5 and 10 µm (Fig. 1C, D). When loaded with higher concentrations of SIM (10^−4^ M and 10^−3^ M), the morphology of the coating showed poor crystallinity (Fig. 1E, F).

The morphology of the Ca-P coating loaded with 10^−2^ M MNZ showed a decreased crystal size, assumed a marked curvature, and became more densely packed (Fig. 1G, H). There was no marked difference in the morphology of the coating when loaded with different concentrations of MNZ (Fig. 1I, J).

SEM observations of the Ca-P coating loaded with 10^−2^ M MNZ and 10^−5^ M SIM together showed that the morphology of the coating was similar to the Ca-P coating loaded with 10^−2^ M MNZ, except that the thickness of the curved crystal increased slightly and the edge of the crystal became blunted (Fig. 1K, L).

### Release kinetics of SIM and MNZ from drug-loaded Ca-P coating

**Figure 2:**
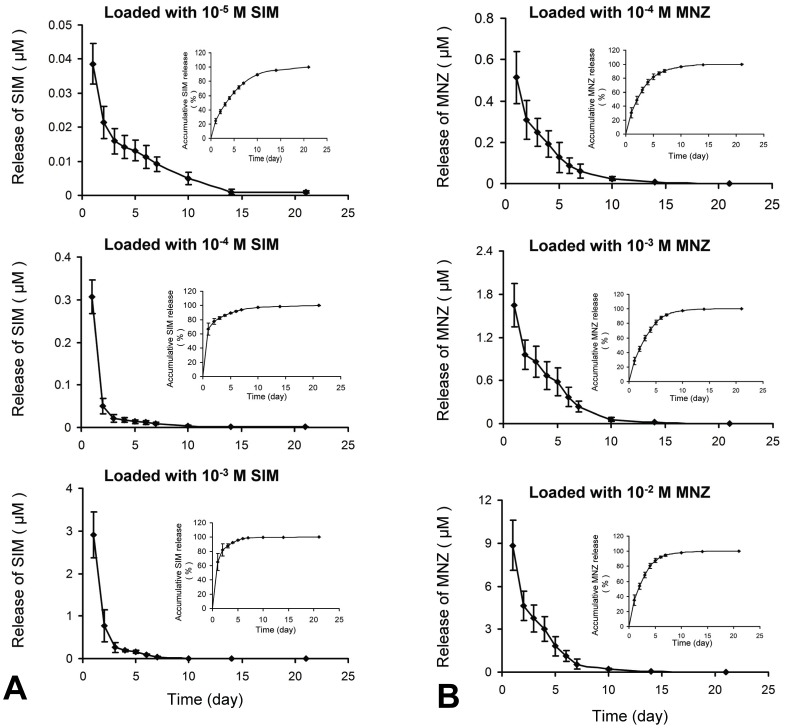
*In vitro* SIM and MNZ release kinetics in PBS from drug-loaded Ca-P coatings. (A) SIM release kinetics. (B) MNZ release kinetics.

When loaded with 10^−5^ M SIM, the coating demonstrated slow-release characteristics without an obvious burst phase, and the concentration of SIM in the culture well remained at 0.01 µM even after 7 days of exposure to PBS. When loaded with higher concentrations of SIM (10^−4^ M and 10^−3^ M), the release kinetics showed a burst phase during the first 24 h (Fig. 2A). When loaded with 10^−3^ M SIM, the burst phase release on the first day surpassed 2 µM.

MNZ release detection showed that MNZ incorporated in the coatings was slowly released as well. However, it was only in the 10^−2^ M group that the release of MNZ could sustain a release level of 3.0 µM (higher than 0.5 µg/mL) after 4 days of exposure to PBS (Fig. 2B).

### Elemental analysis of the drug loaded Ca-P coatings

**Figure 3:**
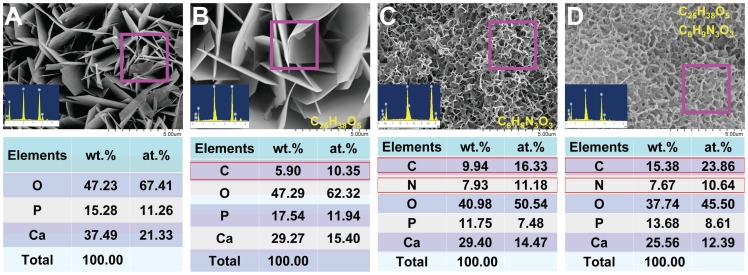
Energy dispersive X-ray spectroscopy (EDS) analysis of the elementary components of the coating. (A) The Ca-P coating was mainly composed of calcium, phosphate and oxygen. (B) When loaded with 10^−5^ M SIM, carbon could also be detected. (C) When loaded with 10^−2^ M MNZ, carbon and nitrogen could also be detected. (D) When loaded with 10^−2^ M MNZ and 10^−5^ M SIM together, the proportion of carbon increased compared with the Ca-P coating loaded with MNZ alone.

EDS analysis of the elementary components of the Ca-P coating showed that the coating was mainly composed of the elements calcium, phosphate and oxygen (Fig. 3A). When loaded with 10^−5^ M SIM (C_25_H_38_O_5_), we detected carbon as well (Fig. 3B). When loaded with 10^−2^ M MNZ (C_6_H_9_N_3_O_3_), we detected carbon and nitrogen, besides the three basic elements of calcium, phosphate and oxygen (Fig. 3C). When loaded with 10^−2^ M MNZ and 10^−5^ M SIM together, we detected carbon and nitrogen, and the proportion of carbon was increased compared with the MNZ-loaded Ca-P coating alone (Fig. 3D).

### Antibacterial capability of the drug-loaded Ca-P coatings

**Figure 4:**
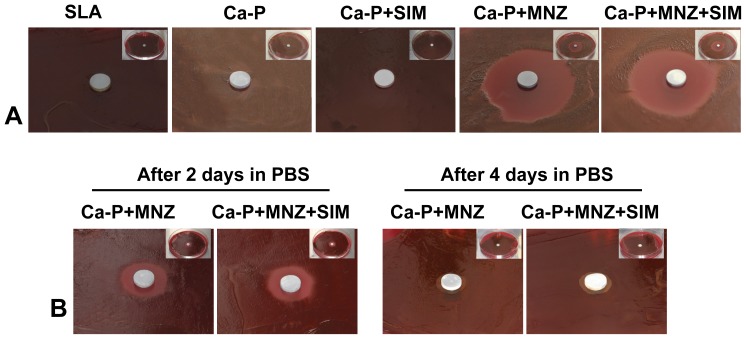
Bacterial inhibition capability of the drug-loaded Ca-P coatings. (A) Inhibition zones formed by different groups of Ti disks. (B) Inhibition zones formed by the Ca-P+MNZ and Ca-P+MNZ+SIM groups after 2 and 4 days exposure to PBS.

Zones of inhibition of bacterial growth were observed in the Ca-P+MNZ and Ca-P+MNZ+SIM groups (Fig. 4A). There was no significant difference (*P*>0.05) in the diameter of the inhibition zones between the two groups (Table 2). No inhibitory effect was observed in the SLA, Ca-P, or Ca-P+SIM groups (Fig. 4A).

**Table 2:** Inhibition zone diameters for different groups of Ti disks.

Group	SLA	Ca-P	Ca-P+SIM	Ca-P+MNZ	Ca-P+MNZ+SIM
**Diameter (mm)**	0	0	0	32.5±4.2	30.0±5.0

After 2 and 4 days of exposure to PBS, the Ca-P+MNZ and Ca-P+MNZ+SIM groups formed relatively smaller inhibition zones (Fig. 4B) and there was no significant difference (*P*>0.05) in the diameter of the inhibition zone between the two groups (Table 3).

**Table 3:** Inhibition zone diameters for the Ca-P+MNZ and Ca-P+MNZ+SIM groups after 2 and 4 days exposure to PBS.

Group	After 2 days in PBS		After 4 days in PBS	
	Ca-P+MNZ	Ca-P+MNZ+SIM	Ca-P+MNZ	Ca-P+MNZ+SIM
**Diameter (mm)**	16.0±3.6 mm	15.5±3.7 mm	10.0±1.0 mm	10.5±0.8 mm

### Effects of drug-loaded Ca-P coatings on cell attachment and proliferation

**Figure 5:**
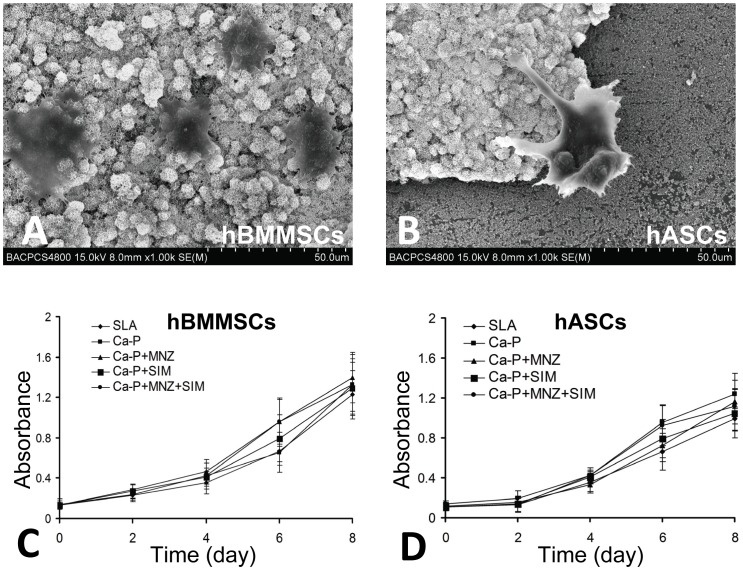
Effects of the drug loaded Ca-P coating on cell attachment and proliferation. (A, B) SEM observation of the cells attached to the bi-functional Ca-P coating. (C, D) Growth curves of the cells cultured on different groups of Ti disks.

SEM observations showed that hBMMSCs and hASCs were able to attach to the surface of the bi-functional Ca-P coatings (Fig. 5A, B). Interestingly, on the border of the coating, the protuberances of cells preferred to stick to the coating surface instead of the Ti surface (Fig. 5B).

The effects of drug-loaded Ca-P coating on the proliferation of hBMMSCs and hASCs are shown as growth curves (Fig. 5C, D). CCK8 assays demonstrated that cell proliferation was not significantly affected by different coating techniques when compared with conventional SLA surface treatment (*P*>0.05 for all time points).

### Effects of drug-loaded Ca-P coatings on the osteogenic differentiation of human MSCs

To determine the pro-osteodifferentiation capability of drug-loaded Ca-P coatings, hBMMSCs and hASCs were seeded onto five groups of Ti disks and induced in osteogenic medium for 7 and 14 days.

**Figure 6:**
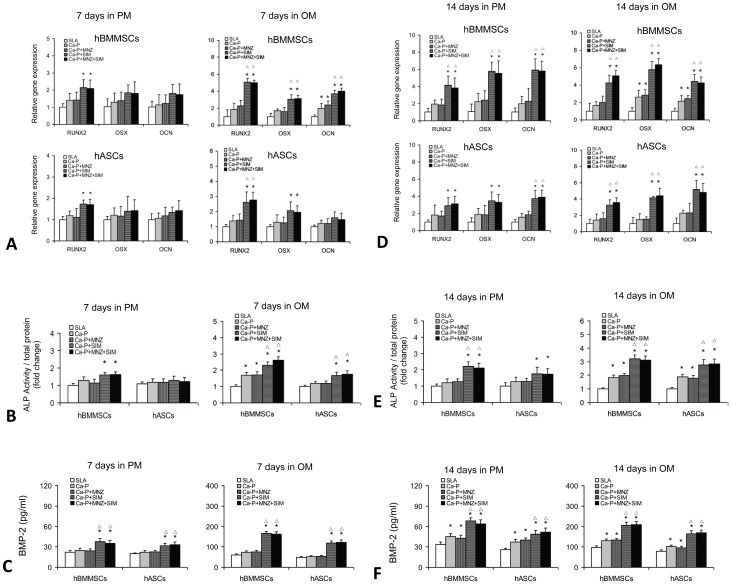
Effects of the drug-loaded Ca-P coating on the osteogenic differentiation of human MSCs. (A) The expression of osteogenic genes in hBMMSCs and hASCs cultured on different groups of Ti disks for 7 days. (B) ALP activity of hBMMSCs and hASCs cultured on different groups of Ti disks for 7 days. (C) The ELISA determination of BMP-2 protein secretion cultured on different groups of Ti disks for 7 days. (D) The expression of osteogenic genes in hBMMSCs and hASCs cultured on different groups of Ti disks for 14 days. (E) ALP activity of hBMMSCs and hASCs cultured on different groups of Ti disks for 14 days. (F) The ELISA determination of BMP-2 protein secretion cultured on different groups of Ti disks for 14 days. *P<0.05 compared with the SLA control group; ^△^ *P<0.05* compared with the Ca-P control group.

After 7 days of culture in osteogenic medium, the expression levels of osteogenic genes (*RUNX2, OSX* and *OCN* in hBMMSCs; *RUNX2* in hASCs) were significantly upregulated in the Ca-P+SIM and Ca-P+MNZ+SIM groups (*P*<0.05) compared with the SLA and Ca-P control groups (Fig. 6A). ALP activity assays (Fig. 6B) showed that the SIM-containing coatings (Ca-P+SIM and Ca-P+MNZ+SIM) significantly increased the ALP activity of both hBMMSCs and hASCs (*P*<0.05) when compared with the control groups of SLA and Ca-P. Interestingly, the ELISA assays showed that, after 7 days of culture in both proliferation medium and osteogenic medium, the level of BMP-2 protein secretion was significantly increased in the Ca-P+SIM and Ca-P+MNZ+SIM groups (*P*<0.05) compared with the SLA and Ca-P control groups (Fig. 6C).

**Figure 7:**
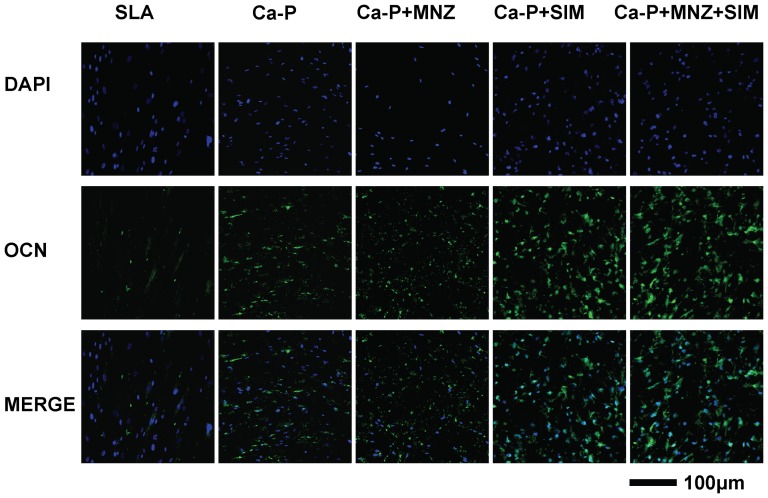
Immunofluorescent staining for OCN in MSCs cultured on different groups of Ti disks for 14 days.

After 14 days of induction, the expression of the osteogenic genes *RUNX2*, *OSX* and *OCN* were significantly upregulated in both hBMMSCs and hASCs in the Ca-P+SIM and Ca-P+MNZ+SIM groups (*P*<0.05) compared with the SLA and Ca-P control groups. More importantly, after 14 days of culture in proliferation medium, the expression of osteogenic genes (*RUNX2*, *OSX* and *OCN* in hBMMSCs; *RUNX2* in hASCs) were significantly upregulated in the Ca-P+SIM and Ca-P+MNZ+SIM groups (*P*<0.05) when compared with the control groups of SLA and Ca-P (Fig. 6D). Similar results were obtained from the ALP activity assays (Fig. 6E) and ELISA assays (Fig. 6F). Moreover, immunofluorescent staining for OCN showed that cells cultured on the SIM-containing coatings (Ca-P+SIM and Ca-P+MNZ+SIM) produced more OCN protein compared with the SLA, Ca-P and Ca-P+MNZ groups (Fig. 7).

## Discussion

Ca-P coating has been shown to improve the performance of metal implants or other bone substitute materials [4], [18]–[20]; however, it does not confer osteoinductivity on the implants. To overcome this problem, we integrated osteoinductive agents into the biomimetic Ca-P coating. SIM, a competitive inhibitor of 3-hydroxy-3-methyl coenzyme A (HMG-CoA) reductase, is a convenient and economical drug which has been widely used to treat hyperlipidemia [12], [21], [22]. By screening over 30,000 natural and artificial compounds, Mundy *et al*. [12] discovered that statins can stimulate the expression of bone morphogenetic protein (BMP)-2 in osteoblasts, and can effectively stimulate bone formation. We and other researchers have further confirmed that SIM can increase the osteogenic capability of MSCs and has therapeutic potential for the treatment of osteoporosis [21], and fracture healing [22].

Here, we applied different concentrations of SIM to a supersaturated Ca-P solution during the second step of the biomimetic Ca-P coating preparation procedure to form a series of SIM-loaded Ca-P coatings. SEM observations determined that only the 10^−5^ M SIM group showed good crystallinity. In SIM release experiments, only the 10^−5^ M SIM group slowly released SIM into the culture well. Moreover, the SIM concentration in the well remained at 0.01 µM after 7 days of exposure to PBS. We previously demonstrated that SIM at 0.01, 0.1 and 1 µM upregulated the expression of osteogenic genes in hASCs, however, high concentrations (>2 µM) of SIM may inhibit cell proliferation [11]. In the 10^−3^ M SIM group, the burst release of SIM on the first day surpassed 2 µM, and therefore 10^−3^ M SIM is not a suitable concentration for loading onto Ca-P coatings. The concentration of SIM released from the 10^−4^ M SIM group was below 2 µM during the course of the experiment; however, in order to maximize the safety of the coating system, we chose the minimum effective SIM concentration (10^−5^ M) for the coating preparation procedure. Cell differentiation experiments demonstrated that the pro-osteodifferentiation capability of the coating was increased when loaded with 10^−5^ M SIM, further confirming that 10^−5^ M is an effective loading concentration for SIM.

Orthopedic implant-associated infections are among the most common complications associated with devices for bone fracture fixation, joint replacement and dental implants. Bacterial colonization and biofilm formation on the implanted device may lead to acute and chronic infection of the underlying bone and the adjacent soft tissues [23]. Prolonged use of antibiotics at higher doses to cure such infections may lead to drug resistance, systemic and local toxicity, and could potentially compromise bone growth and immune surveillance. Such limitations have prompted the development of alternative prophylactic and therapeutic methods to prevent and treat infection, including better physiochemical modifications and more efficacious coatings on the implant surface [1].

MNZ is a widely used antibiotic with selective toxicity to microaerophilic and anaerobic bacteria [13], [14], [24]. It functions by inhibiting bacterial DNA synthesis, resulting in cell death [25]. In this study, MNZ was loaded into the biomimetic Ca-P coating as a local antibiotic factor. Similarly to SIM, various concentrations of MNZ were applied to a supersaturated Ca-P solution during the second step of the Ca-P coating preparation procedure to form a series of MNZ-loaded Ca-P coatings.

The minimum inhibitory concentration (MIC) range of MNZ against microaerophilic and anaerobic bacteria is 0.06 to 1 µg/mL and the MIC_90_ is 0.5 µg/mL [13]. We observed slow release of MNZ at various concentrations; however, only the 10^−2^ M MNZ group sustained an MNZ concentration of 3 µM in the culture well (>0.5 µg/mL) after 4 days of exposure to PBS during the MNZ release experiments. Therefore, we chose 10^−2^ M as a suitable final MNZ concentration in the second step of the coating preparation procedure.

To construct a novel bi-functional Ca-P coating, we integrated 10^−2^ M MNZ and 10^−5^ M SIM together into biomimetic Ca-P coatings. To test these coatings *in vitro*, human MSCs and *P. gingivalis* were used to assess the pro-osteodifferentiation and antibacterial capabilities of this bi-functional coating.

Zone of inhibition experiments confirmed that the growth of *P. gingivalis* was inhibited by coatings containing MNZ (Ca-P+MNZ+SIM and Ca-P+MNZ). Moreover, the results also proved that the presence of SIM did not influence the biological effects of MNZ. Interestingly, we also found that the MNZ-loaded Ca-P coatings retained their antibacterial effects even after 2 and 4 days of exposure to PBS. This suggests that the bi-functional coating (Ca-P+MNZ+SIM) prepared in this study could maintain its antibacterial capability for a certain period of time in a liquid environment similar to *in vivo* conditions. The initial post-operative stage is a dangerous stage for patients receiving orthopedic implants, due to the increased risk of infection caused by pathogenic microorganisms, and prophylactic antibiotic application is a simple and practical way to circumvent this problem [26]–[29]. The systemic application of antibiotics has several drawbacks as outlined above, which can be averted by the local release of MNZ from the bi-functional coating over several days.

Cell proliferation experiments demonstrated that a bi-functional coating loaded with specific concentrations of MNZ and SIM had negligible adverse effects on the proliferation of human MSCs. Furthermore, cell differentiation experiments that measured ALP activity, BMP-2 protein secretion, OCN protein expression and osteogenic gene expression suggested that SIM-loaded coatings could markedly stimulate the osteogenic differentiation of hBMMSCs and hASCs, even in proliferation medium. These results are encouraging, as hBMMSCs and hASCs have been considered as effective sources of adult MSCs and can be applied in stem cell-based therapies and bone tissue engineering applications [30]–[37]. As MSCs are initially recruited to the implant surface when it is surgically implanted, the bi-functional coating presented here could enhance osseointegration by direct inducing the recruited MSCs. Also, the osseointegration of implants is accelerated if the implant is surrounded by a sheet of hBMMSCs [38]; therefore, this bi-functional coating could further improve osseointegration when combined with the cell sheet technique. Furthermore, this coating technique can be easily applied not only to the surface of metal implants, but also to the internal and external surfaces of polyporous bone scaffold materials [5] to improve their antibacterial and pro-osteodifferentiation capabilities.

There were some limitations to our study. Firstly, we cannot accurately measure the slow-release of SIM and MNZ from the bi-functional Ca-P coating, as SIM and MNZ are released together into the PBS and cannot be completely separated by spectrophotometry. However, the results of *in vitro* experiments showed that the antibiotic capabilities of bi-functional coatings were similar to the Ca-P+MNZ group, and the pro-osteodifferentiation capacity of the bi-functional coating was consistent with the Ca-P+SIM group. These results suggest that simultaneously loading SIM and MNZ into Ca-P coatings will not affect their function and release. Secondly, further *in vivo* investigations should be done in the future to fully reveal the performance of this bi-functional Ca-P coating.

## Conclusions

In this study, we have successfully integrated simvastatin and metronidazole into a Ca-P coating for titanium surface, and explored the pro-osteodifferentiation and antibacterial capabilities of this coating. We demonstrated the controlled release of both simvastatin and metronidazole from the coating, along with increased osteogenic cell differentiation and the inhibition of bacterial growth. Considering the safety, stability and low cost of simvastatin and metronidazole, this bi-functional Ca-P coating technique represents a promising method to improve the performance of metal implants or other bone substitute materials, and can theoretically be easily translated to clinical applications. However, further characterization of the bi-functional coatings described above is necessary, as well as *in vivo* studies to properly assess the therapeutic potential of this technology.
